# Association between the Hypomethylation of Osteopontin and Integrin β3 Promoters and Vascular Smooth Muscle Cell Phenotype Switching in Great Saphenous Varicose Veins

**DOI:** 10.3390/ijms151018747

**Published:** 2014-10-17

**Authors:** Han Jiang, Yu Lun, Xiaoyu Wu, Qian Xia, Xiaoyu Zhang, Shijie Xin, Jian Zhang

**Affiliations:** Department of Vascular and Thyroid Surgery, the First Affiliated Hospital of China Medical University, Shenyang 110001, China; E-Mails: cmu_jianghan@126.com (H.J.); cmu_lunyu@163.com (Y.L.); cmu_wuxiaoyu@163.com (X.W.); cmu_xiaqian@163.com (Q.X.); cmu_zhangxiaoyu@163.com (X.Z.); cmu_xinshijie@163.com (S.X.)

**Keywords:** varicosity, vascular smooth muscle cells, phenotype switching, osteopontin, integrin β3, DNA methylation

## Abstract

Lower extremity varicose veins are a common condition in vascular surgery and proliferation of vascular smooth muscle cells (VSMCs) in the intima is a significant pathological feature of varicosity. However, the pathogenesis of varicose veins is not fully understood. Osteopontin (OPN) could promote the migration and adhesion of VSMCs through the cell surface receptor integrin β3 and the cooperation of OPN and integrin β3 is involved in many vascular diseases. However, the role of OPN and integrin β3 in varicosity remains unclear. In the current study, we found that the methylation levels in the promoter regions of OPN and integrin β3 genes in the VSMCs of varicose veins are reduced and the protein expression of OPN and integrin β3 are increased, compared with normal veins. Furthermore, it was observed that VSMCs in the neointima of varicose veins were transformed into the synthetic phenotype. Collectively, hypomethylation of the promoter regions for OPN and integrin β3 genes may increase the expression of these genes in varicosity, which is closely related to VSMC phenotype switching. Hypomethylation of the promoter regions for OPN and integrin β3 genes may be a key factor in the pathogenesis of varicosity.

## 1. Introduction

Lower extremity venous insufficiency is a common condition in vascular surgery, with approximately 25% of the population having lower extremity varicose veins [1]. Most of these are varicosis of the great saphenous vein. The pathogenesis of varicosis is not fully understood. Vascular remodeling is closely related to changes in gene expression, physical and chemical vascular damage and stimulation of metabolic and vasoactive substances [2,3]. Proliferation of vascular smooth muscle cells (VSMCs) in the intima is a significant pathological feature of varicosity [4]. Phenotype switching of VSMCs is the cytological basis for vascular remodeling. Under pathological conditions, VSMCs exhibit a phenotypic change characterized by loss of contractility and abnormal proliferation, migration and matrix secretion. This synthetic phenotype plays an active role in the repair of vascular damage and is also related to the pathogenicity of several cardiovascular diseases [5]. Epigenetics is defined as genome properties that are not explained by the primary DNA sequence, but are, instead, caused by modifications of the DNA and/or its associated proteins [6]. Epigenetics includes the study of DNA methylation, histone modifications, and the interaction of microRNAs (miRNAs) with the genome. DNA methylation is a well-known example of an epigenetic mechanism [7], which plays an important role in maintaining the normal structure of the DNA, chromatin stability, and X-chromatin inactivation [8,9]. DNA methylation plays an important role in regulating gene expression and a negative correlation exists between the degree of DNA methylation and gene expression. Methylation status of DNA is reportedly involved in the regulation of pathological changes in arterial atherosclerosis, diabetes, inflammation and hypertension. Recently, DNA methylation has become the focus of research in many fields [10,11,12]. Epigenetic regulation of VSMC phenotype switching occurs mainly through the regulation of phenotype markers [13]. However, little is known about how to maintain the contractile phenotype of VSMCs under physiological conditions. In addition, whether abnormal epigenetic regulation participates in the development of varicose veins has not been reported. Osteopontin (OPN) is a multifunctional phosphoprotein secreted by many cells. OPN is a biomarker of VSMC phenotype switching and is strongly expressed by synthetic VSMCs [14,15]. The anti-inflammatory, injury repair and vascular remodeling effects of OPN have aroused research interest [15,16]. OPN is closely associated with VSMC proliferation and migration [16,17] and has emerged as a key factor in both vascular remodeling and development of restenosis [18]. Integrin β3 is one of the OPN receptors found in a wide variety of cells. This class of adhesion receptors could mediate cell–cell and cell–extracellular matrix (ECM) interactions. Such interactions are important for tissue integrity, cellular migration, cell survival, adhesion and differentiation [19,20,21]. Integrin β3 plays an important role in regulating VSMC migration and proliferation in vivo and in vitro [20,21]. OPN could promote the migration and adhesion of VSMCs through the integrin β3 cell surface receptor [22]. In this study, we examined the methylation status in the promoter regions for OPN and integrin β3 genes, the expression level of OPN and integrin β3 in varicose vein specimens, and the ultrastructural changes of VSMCs in the neointima of varicose veins. We also investigated the relationship between the methylation status of the promoter regions for OPN and integrin β3 and the phenotype switching of VSMCs in varicose veins. Collectively, we provide experimental evidence for the pathogenesis of varicose veins.

## 2. Results and Discussion

### 2.1. Observations of the Vein Wall by Hematoxylin and Eosin (H&E) Staining and Ultrastructure Change of Vascular Smooth Muscle Cells (VSMCs) by Transmission Electron Microscope (TEM)

**Figure 1:**
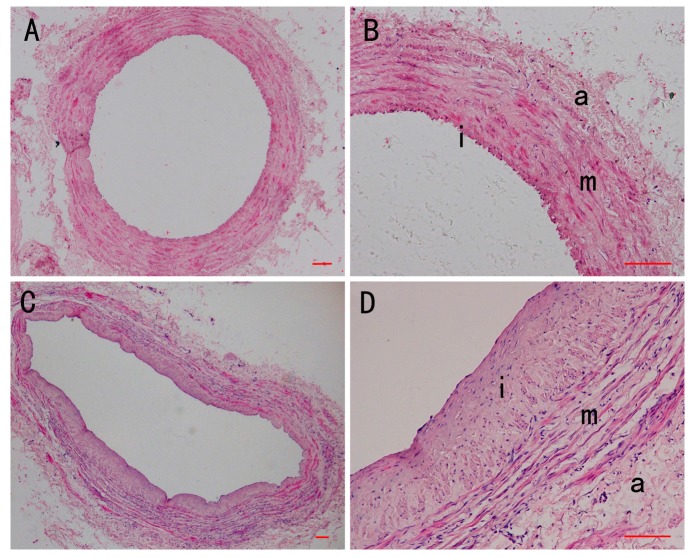
Histological structure of the vein wall (H&E staining). (**A**) Section of normal vein shows the regularity of wall and the normal thickness of the layers; (**B**) A higher magnification of the previous section shows the intima (i), media (m) and adventitia (a); (**C**) Section of varicose vein showing the irregularity of the wall and the intima hypertrophy; (**D**) A higher magnification of the previous section shows the neointima (i), abnormal arrangement of VSMC in media (m) and adventitia (a). Scale bar = 200 μm.

**Figure 2:**
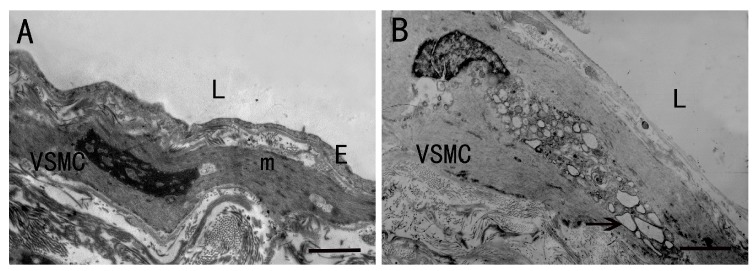
The tunica intima of normal vein and varicose veins. (**A**) Normal vein, lumen (L) and endothelial cells (E) cytoplasm of intima vascular smooth muscle cells (VSMCs) filled with myofilaments (m); (**B**) Varicose vein, losing all the endothelial cells, lumen (L). → indicates many rough endoplasmic reticulum of intima VSMC. Scale bar = 2 μm.

In the normal vein group, the thickness of the vein wall was regular and the wall framework consisted of three distinct concentric layers. The intima of the vein wall, lined by endothelial cells, rests on well-defined subendothelial connective tissue. The media of the vein wall contained circular bundles of VSMCs. The adventitia contained clustered longitudinal VSMCs and ECM (Figure 1A,B). According to the study of Pamela *et al.* [4], we defined the longitudinally oriented VSMC layer adjacent to lumen as the intima and the circular oriented VSMC layer followed intima as the media in varicose vein. In the varicose vein group, the thickness of vein wall was not uniform. Dilatation of the lumen and increased intimal thickness were both observed. There are a number of longitudinally oriented VSMCs in the neointima of varicose veins. The neointima was folded, with partial desquamation of its endothelial cells. The organization of the three layers was greatly disrupted consistent with previous studies [23], the smooth muscle bundles had lost their longitudinal or circular orientation and an accumulation of fibrous tissue interrupted the regular pattern of smooth muscle bundles (Figure 1C,D). The results suggest that the vein wall of varicose vein lost its normal structure. To observe the ultrastructure change of VSMCs in intima of veins, TEM was employed. Sections from the normal vein group showed endothelial cells and VSMC in the intima, and cytoplasm of intima VSMC is filled with myofilaments (Figure 2A). In sections from the varicose vein group, complete disorganization of the neointima and loss of endothelial cells were observed. The shape of VSMC nuclei is irregular, with karyotheca of partial rupture. In cytoplasm, many dilated rough endoplasmic reticulum and golgi apparatus could be observed (Figure 2B). Consistent with the study of Thyberg *et al.* [24], our results suggest that the VSMC in the neointima of varicose vein have the characteristics of synthetic phenotype.

### 2.2. Immunohistochemical Analysis of Osteopontin (OPN), Integrin β3 and Smooth Muscle α-Actin (SMA) and Western Blot for OPN and Integrin β3

**Figure 3:**
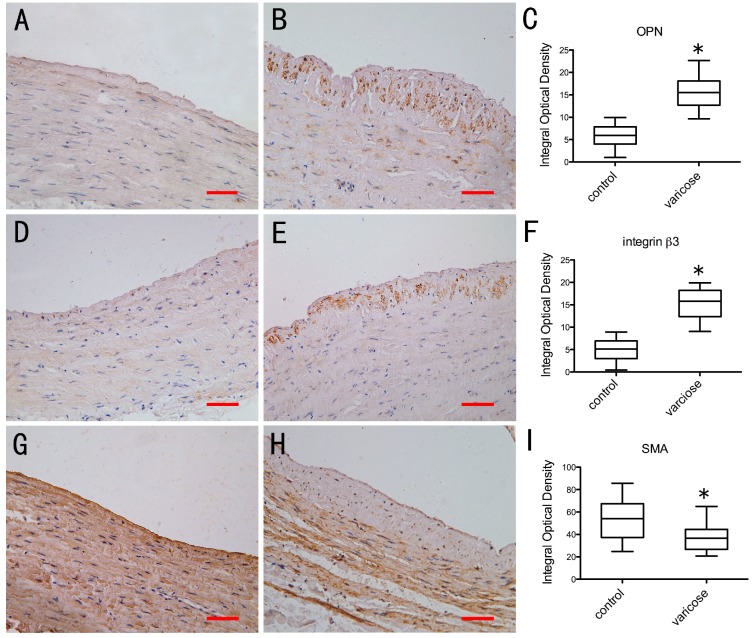
Immunostaining of Osteopontin (OPN), integrin β3 and Smooth Muscle α-Actin (SMA) in vein specimens. (**A**) immunostaining of OPN in normal vein; (**B**) immunostaining of OPN in varicose vein; (**D**) immunostaining of integrin β3 in normal vein; (**E**) immunostaining of integrin β3 in varicose vein; (**G**) immunostaining of SMA in normal vein; (**H**) immunostaining of SMA in varicose vein; (**C**,**F**,**I**) Quantitative analysis of the difference of OPN, integrin β3 and SMA expression in normal vein and varicose vein. Data are presented as medians and inter-quartile ranges (* p < 0.0001 *vs.* Normal veins). Scale bar = 50 μm.

**Figure 4:**
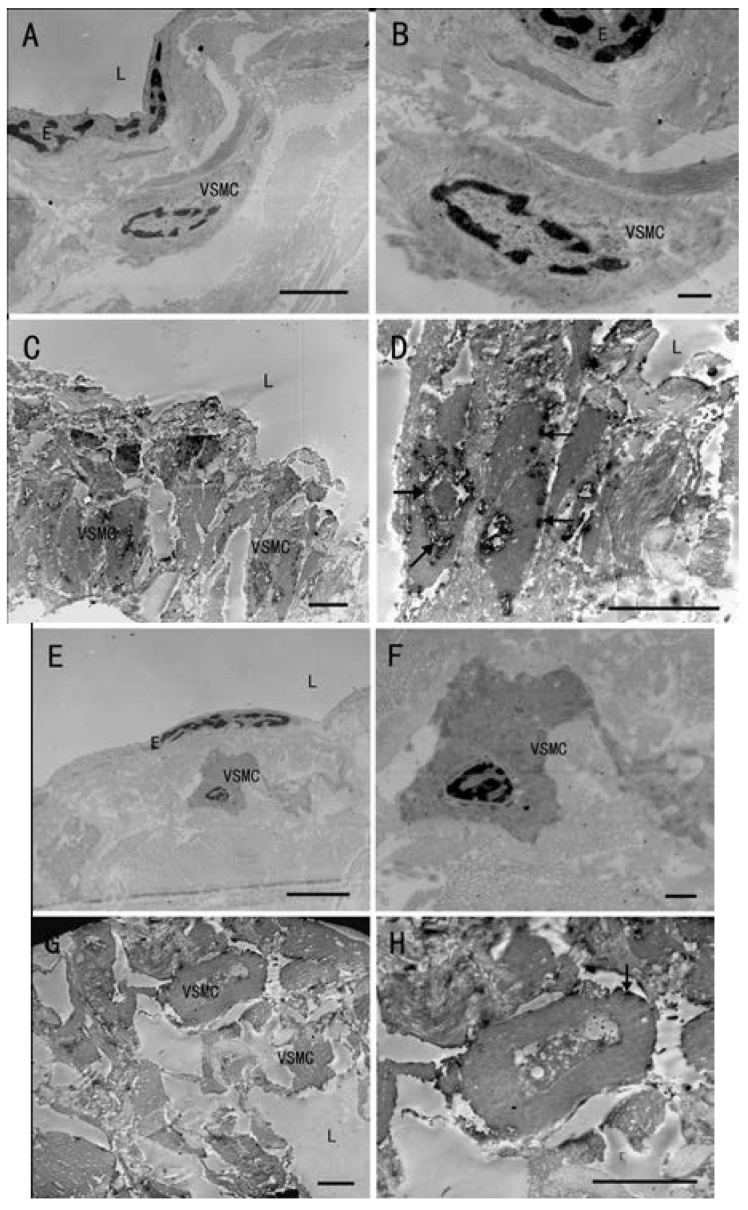
Expression of OPN and integrin β3 as detected by TEM. Normal vein with rare positive OPN (**A**,**B**) or integrin β3 (**E**,**F**) electron-dense black granules in VSMC. Varicose veins with OPN electron-dense black granules (**C**,**D**) located on the nuclear membrane (→), rough endoplasmic reticulum (↗) and cytomembrane (←) of intimal VSMC. Varicose veins with integrin β3 electron-dense black granules (**G**,**H**) located on the cytomembrane (↓) of intimal VSMC. Lumen (L). Endothelial cell (E). Scale bar = 5 μm.

**Figure 5:**
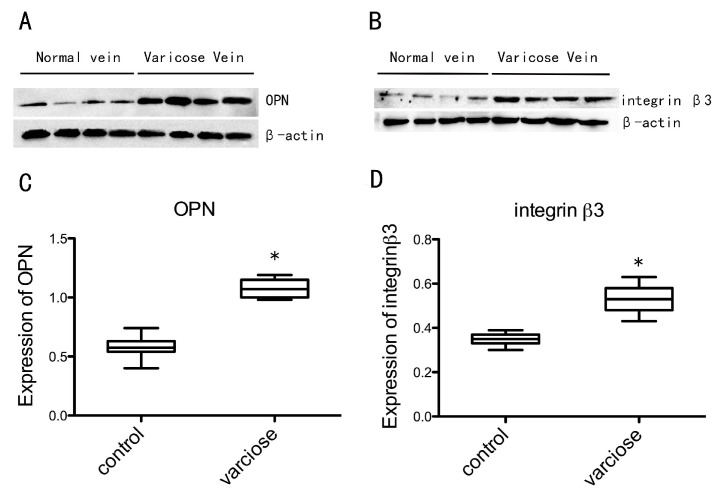
Western blots showing the expression of OPN and integrin β3 in varicose veins compared with normal veins. Expression of OPN (**A**) and integrin β3 (**B**) in the intima of vein specimens; Differences in OPN (**C**) and integrin β3 (**D**) expression in the intima of vein specimens. Data are presented as medians and inter-quartile ranges (* p <0.0001 *vs.* Normal veins).

With immunohistochemical staining, positive cells stained brown yellow. In the normal vein group, only a few VSMCs in the intima region were found to be OPN-positive (Figure 3A) or integrin β3-positive (Figure 3D). A number of Smooth Muscle α-Actin (SMA) positive VSMCs could be found in media region (Figure 3G). However, in the varicose vein group, OPN-positive (Figure 3B) and integrin β3-positive (Figure 3E) VSMCs were significantly more numerous, mainly in the neointimal region. In varicose vein, SMA positive VSMCs were rarely detected in neointima region. In media region of varicose vein, SMA positive VSMCs could be observed (Figure 3H). We recorded the optical density (OD) of positive cells in each field to evaluate the average OD, and analyzed the results with the MetaMorph/DPIO/BX41 morphology image analysis system. There were statistically significant differences between the two groups in positive expression of OPN, integrin β3 and SMA (Figure 3C,F,I; *p* < 0.0001). There are few contractile phenotype but a large number of synthetic phenotype VSMCs in neointima region of varicose veins. In order to determine subcellular localization of OPN and integrin β3, immunohistochemical analysis of OPN and integrin β3 expression with TEM was performed. The positive expression of OPN and integrin β3 produced electron-dense black granules. In the intima of the normal vein group, there were almost no VSMCs positive for OPN (Figure 4A,B) or for integrin β3 (Figure 4E,F). In the neointima of the varicose vein group, expression of OPN and integrin β3 could be detected. The granules produced by OPN were located on the nuclear membrane, the rough endoplasmic reticulum and cytomembrane of VSMCs in the neointima (Figure 4C,D). The granules produced by integrin β3 were located on cytomembrane of the VSMCs in the neointima (Figure 4G,H). The results suggest that presence of both markers on the membrane of VSMCs in the neointima. The OPN, integrin β3 and β-actin proteins were detected at 35, 87 and 43 KD, respectively (Figure 5A,B). Compared with the normal vein group, the density of OPN and integrin β3 bands in the varicose vein group was significantly higher (Figure 5C,D; *p* < 0.0001), which was consistent to the results from immunohistochemical analysis. In Comparison to the normal vein group, the expression of OPN and integrin β3 in the varicose vein group was significantly increased. Both of OPN and integrin β3 are closely associated with the process of VSMC phenotype switching in neointima of varicose veins.

### 2.3. DNA Hypomethylation in the Promoter Regions of OPN and Integrin β3

**Figure 6:**
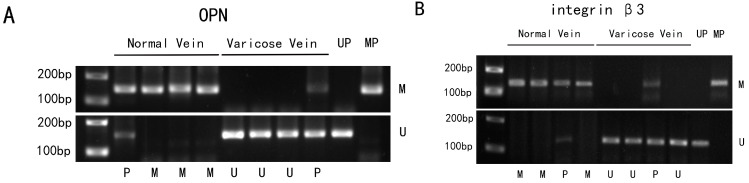
Differences in OPN and integrin β3 gene promoter methylation in vein specimens. (**A**) Methylation specific PCR (MSP) analysis of OPN gene promoter methylation in vein specimens; (**B**) MSP analysis of integrin β3 gene promoter methylation in vein specimens. Methylation, unmethylation, unmethylation positive control, methylation positive control and partial methylation are simply labeled as M, U, UP, MP, and P, respectively.

To identify whether high expression of OPN and integrin β3 resulted from DNA hypomethylation, we examined the methylation of OPN and integrin β3 genes in the DNA of vein specimen. Methylation specific PCR (MSP) showed that both unmethylated and methylated bands existed in normal and varicose veins (Figure 6A,B), but the unmethylated bands did not appear in the positive control tissues. The DNA methylation level was lower in varicose veins group compared with the normal veins group. DNA hypomethylation of OPN occurred in 62.2% (28/45) of varicose veins and 16.6% (5/30) of normal veins. DNA hypomethylation of integrin β3 occurred in 55.6% (25/45) of varicose veins and 20% (6/30) of normal veins. The differences in methylation of OPN (*p <* 0.000) and integrin β3 (*p <* 0.001) between normal veins and varicose veins were significant (Table 1). According to our results, DNA hypomethylation may participate in the process of regulating OPN and integrin β3 in varicose vein.

**Table 1:** Methylation levels of OPN and integrinβ3 in different vein groups.

Heading	Patients (*n*, *N* = 75)	OPN Methylation			*P* Value	Integrin β3 Methylation			*P* Value
		M (%)	P (%)	U (%)		M (%)	P (%)	U (%)	
**Normal Vein**	30	17 (56.7)	8 (26.7)	5 (16.6)	0.000	19 (63.3)	5 (16.7)	6 (20)	0.001
**Varicose Vein**	45	8 (17.8)	9 (20)	28 (62.2)		10 (22.2)	10 (22.2)	25 (55.6)	

### 2.4. Discussion

Lower limb varicose veins are a common peripheral vascular disease, and many factors have been implicated in their etiology. Dilatation of the vein, increased diameter of the lumen, and hypertrophy of the vein wall and intima are important characteristics of the pathological morphology in varicose veins [25]. However, there is ample evidence that the defect results from intimal changes in the walls of lower limb veins [23,26]. VSMCs are the main components of the media of the vascular wall. The phenotype of VSMC includes contractile phenotype and synthetic phenotype. Unlike other muscle cells, contractile phenotype VSMCs do not terminally differentiate. Under normal conditions, the contractile phenotype VSMCs in adult animals are highly specialized cells, functioning to regulate blood vessel diameter, blood pressure and blood flow distribution. However, these cells display remarkable plasticity and undergo profound changes in phenotype in response to vascular injury or disease, a process referred to as phenotypic switching. As part of the repair process in response to injury, contractile phenotype of VSMCs could convert into synthetic phenotype, which have stronger proliferative capacity. In addition, they could synthesize and secrete large amounts of ECM. Phenotype switching occurs as the local environment changes or vascular lesions occur, such as hypertension, vascular grafts, arteriosclerosis, mechanical stimulation and cytokines release [13,14,27]. Khan, A.A. *et al.*, showed that the intima hypertrophy of varicose vein could result from the overall increase of smooth muscle cells [19]. In varicose veins, rough endoplasmic reticulum and golgi complex could be observed in VSMCs of neointima indicating that synthesis and secretory functions of VSMCs may be enhanced. The VSMCs of neointima in varicose veins were markedly altered, which may be the phenotype switching of VSMCs.

SMA is a characteristic cytoskeletal protein of VSMC and plays an important role in cell contraction [28]. SMA is found progressively increased in the contractile phenotype of VSMC. Conversely, when the VSMC undergoes inappropriate proliferation or is converted into synthetic phenotype, SMA is progressively lost [29]. Therefore, SMA is considered as a biomarker of contractile phenotype VSMC. OPN is another biomarker of synthetic phenotype VSMCs. Expression of OPN is rare in normal vein walls, but several cytokines can induce VSMC proliferation, concurrent with significant increase of OPN expression [30]. In general, VSMC phenotypic switching is characterized by markedly increased expression of synthesis phenotypic markers, with increased VSMC proliferation, migration and synthesis of ECM components required for vascular repair [13,31]. In our study, a large number of cells in the tunica media region of normal vein are found to be SMA positive. However, OPN positive cells are rare. In the mean time, we found intense expression of OPN and rare expression of SMA in the VSMCs of neointima in varicose veins compared with normal veins, indicating transformation of the VSMCs from the contractile phenotype to the synthetic phenotype.

VSMC proliferation is associated with varicose veins [32]. Proliferation of VSMC is the common pathological basis in hypertension, atherosclerosis and restenosis after angioplasty. Some studies have shown that variety of vasoactive peptide are involved in the proliferation of VSMC, such as angiotensin and endothelin [33,34]. VSMCs change phenotype, proliferate and migrate as part of the injury repair process [35]. The mRNA expression of OPN in VSMC significantly correlates with cell migration ability, and under certain conditions, the correlation has a dose-response relationship [36]. The lack of OPN expression in VSMCs can significantly reduce cell adhesion and migration capabilities [37]. Under pathological conditions, such as human atherosclerotic lesions and restenosis, the expression of OPN is significantly increased in the VSMCs of the neointima [38,39,40]. Study of Coskund *et al.* [41] indicated concentration of OPN in plasma has a positive correlation between the severity of the lesion.

The expression of OPN DNA of has cell specificity and is affected by various cytokines. Several biological effects of OPN are associated with the interaction of different receptors, which can directly or indirectly activate various signal transduction pathways. Integrin β3 is one of the known OPN receptors, which plays an important role in regulating VSMC migration and proliferation. OPN could promote the migration and adhesion of VSMCs through the integrin β3. In the current study, we found that integrin β3 was strongly expressed in the VSMCs of neointima compared with those of normal veins. We also found a positive correlation between the expression of integrin β3 and OPN. As a cytokine, OPN can increase integrin β3 expression and induce rapid and transient focal adhesion kinase (FAK) phosphorylation [22]. Integrin β3-FAK signaling is involved in VSMC proliferation and migration [22]. In the pathogenesis of varicose veins, interaction of OPN and integrin β3 on the cell membrane may activate integrin β3-FAK signaling directly or indirectly. The presence of both markers on the membrane may be associated with phenotype switching of VSMCs in neointima of varicose veins.

DNA methylation is an important epigenetic mechanism in the maintenance of normal DNA structure, contributing to chromosomal stability and normal gene expression. It exists widely in the eukaryotic cells, and is one of the most common forms of DNA covalent modification [42,43]. There has been recent progress in the study of DNA methylation in atherosclerosis, abdominal aortic aneurysm and other cardiovascular diseases [44,45,46] and DNA methylation has become important in epigenetic research. In humans and other mammals, this modification occurs mostly on cytosine residues in CpG islands, characterized by a high density of CG dinucleotides [47,48,49]. Methylation of promoter regions and other distal regulatory components prevents the interaction of transcription factor complexes and DNA, thereby inhibiting gene expression [44]. DNA methylation plays an important role in regulating gene expression. There is a negative correlation between the degree of DNA methylation and gene expression. In the process of tumor formation and development, patterns of DNA methylation change, including decreased methylation levels of the genome and elevated methylation levels in gene promoter regions, which activate oncogenes and inhibit tumor suppressor genes. In the pathological process of atherosclerosis, VSMCs first convert to the synthetic type and migrate from the media into the intima, where they participate in the generation of artery plaque. This process is similar to tumor cell transformation, in which tumor cell generation is usually accompanied by integral hypomethylation of genome DNA [50]. In this study, we found that the promoter regions for OPN and integrin β3 genes in the VSMCs in neointima were hypomethylated compared with those in normal veins, and the expression of OPN and integrin β3 was higher in the VSMCs of neointima. Thus, we identified a negative correlation between methylation levels of the gene promoter regions and expression of OPN and integrin β3. These results indicate that decreased methylation level of OPN and integrin β3 is involved in the intimal thickening of varicosity.

Based on these results, we speculate that hypomethylation of the promoter regions of OPN and integrin β3 genes can increase the expression of OPN and integrin β3 in varicosity. The interaction of OPN and integrin β3 on the cell membrane of VSMCs may activate integrin β3–FAK or other signal pathways, causing VSMCs to transform into the synthetic phenotype and to participate in abnormal proliferation and migration. The hypomethylation of these promoter regions are possibly associated with VSMC phenotype switching and plays a key role in neointima formation of varicosity.

## 3. Experimental Section

### 3.1. Patients and Specimens

The study was approved by the institutional review board of the China Medical University (Project identification code: [2014] 63, Date: 10 March 2014) and a consent form was signed by each participating patient. Varicose vein specimens were collected from 45 patients who had a full history, preoperative physical examination and whole leg duplex mapping. All patients underwent high ligation of the saphenofemoral junction, excision of the specimens, stripping down to the knee and multiple stab avulsions of distal calf varicosities. Normal veins were obtained from 30 individuals who had no clinical evidence of chronic venous insufficiency in either lower limb, and who underwent aortocoronary bypass grafting (Table 2). Specimens were taken from each vein, approximately 3–4 cm from the saphenofemoral junction.

**Table 2:** Information of patients in different groups.

Patient Demographics					
		Normal Vein		Varicose Vein	
No. of Patients		30		45	
Age (year)		57 ± 8		46 ± 5	
Gender (M/F)		17/13		19/26	
No. of CEAP ^a^		0		45	
C2		0		16		
C3		0		17		
C4		0		12		
Diabetes		5		8		
Smoker		6		9		

^a^ CEAP indicates the clinical, aetiological, anatomical and pathological classification for lower extremity varicose veins.

### 3.2. Hislogical Analysis

The vein fragments were washed in phosphate-buffered saline (PBS) (Boster, Wuhan, China), opened transversely and fixed in 4% paraformaldehyde (Beijing chemical works, Beijing, China) for 24 h. Then the samples were embedded in paraffin to obtain transverse sections. Serial sections were cut at 4 μm and prepared for H&E staining. For either normal vein (*n* = 30) or varicose vein (*n* = 45) group two sections per patient were cut. For immunochemical staining, four sections per patient were cut from either normal (*n* = 30) or varicose vein (*n* = 45) group. The sections were treated with 3% hydrogen peroxide/methanol (Mai Xin_Bio, Fuzhou, China), followed by incubating with rabbit monoclonal antibody against OPN (ab91655,1:100; Abcam, Cambridge, UK), mouse monoclonal antibody against integrin β3 (ab125717, 1:50; Abcam, Cambridge, UK) and α-SM-actin (14395-1-AP, 1:100: proteintech, Chicago, IL, USA) in 2% bovine serum albumin PBS overnight at 4 °C. After being washed with PBS, the sections were incubated with anti-rabbit or anti-mouse IgG-HRP antibody (Boster, Wuhan, China) for 0.5 h at 37 °C. Finally, 3,3'-diaminobenzidine (DAB) (Mai Xin_Bio, Fuzhou, China) was used as a chromogen for 10 min until the brown yellow color appeared. Sections were then dehydrated and mounted with mounting media. To assess nonspecific staining, a few sections in each experiment were incubated in PBS without primary antibody. One slide was randomly selected from every specimen in the normal vein and the varicose vein groups. Five visual fields were randomly selected on each slide. We recorded the optical density (OD) of positive cells in each field to evaluate the average OD, and analyzed the results with the MetaMorph/DPIO/BX41 morphology image analysis system.

### 3.3. Transmission Electron Microscopy

Each group of dissected great saphenous vein samples were separated into pieces and fixed with 2.5% glutaraldehyde (Sinopharm Chemical Reagent Co., Ltd., Shanghai, China) at 4 °C. The pieces were postfixed in 1% osmium tetroxide (Electron Microscopy Sciences, Hatfield, UK) for 2 h at 4 °C, rinsed in 0.1 M PBS (pH 7.4) several times, dehydrated in a gradient series (20%–100%) of ethanol (Beijing chemical works, Beijing, China) and then followed by 100% acetone (Beijing chemical works, Beijing, China). The resultant samples were then infiltrated with Epon 812 (SERVA, New York, NY, USA) and finally polymerized in pure Epon 812 at 65 °C for 72 h. Semi-thin sections (1 μm) stained with toluidine blue (Beijing chemical works, Beijing, China) were obtained for observation. Ten serial ultra-thin sections (70 nm) per patient were obtained for a total of 750 sections in normal (*n* = 30) and varicose vein (*n* = 45) group. The resultant ultra-thin sections were collected on copper grids. To assess the VSMCs, sections were stained with uranyl acetate (Kojima Chemicals Co., Ltd., Tokyo, Japan) and lead citrate (Alfa Aesar, Ward Hill, MA, USA). Changes in ultrastructure were examined with TEM (JEM-1200EX, JEOL, Tokyo, Japan). For immunohistochemical analysis by TEM, sections (4 μm) were underwent immunohistochemical stained with anti-OPN and integrin β3 antibodies and evaluated by chromogenic reaction. After being washed with PBS, the sections were fixed in 1% osmium tetroxide (Electron Microscopy Sciences, Hatfield, UK) at 4 °C for 20 min. Then they were washed in PBS several times, dehydrated in a gradient series (20%–100%) of ethanol and followed by100% acetone. The resultant samples were then infiltrated with Epon 812 and finally polymerized in pure Epon 812 at 65 °C for 48 h. Ten serial ultra-thin sections (70 nm) per patient were obtained for a total of 1500 sections in normal (*n* = 30) and varicose vein (*n* = 45) group, which were stained anti-OPN and integrin β3 antibodies. The resultant ultra-thin sections were collected on copper grids and stained with 4% uranyl acetate. The positive cells were examined by TEM (JEM-1200EX, JEOL, Tokyo, Japan).

### 3.4. Protein Isolation and Western Blot

Total protein was extracted from tissue specimens through homogenization, ultrasonic dispersion and centrifugation. Extracted proteins were quantified and resuspended in the samples buffer containing 200 mM tris-buffered saline (TBS) (Boster, Wuhan, China), pH 7.5, 4% sodium dodecyl sulfate (SDS) (Boster, Wuhan, China), 20% glycerol (Sunshine Biotechnology Co., Ltd., Nanjing, China), 10% β-mercaptoethanol (Amresco, Solon, OH, USA) and boiled for 3 min. The protein fraction (50 μg/lane) were loaded onto 12% sodium dodecyl sulfate polyacrylamide gels for electrophoresis and transferred to polyvinylidene difluoride membranes (Millipore Corp., Billerica, MA, USA). The membrane was blocked for 120 min with blocking buffer (5% skim milk (Boster, Wuhan, China) in 50 mM Tris–HCl (Boster, Wuhan, China), 200 mM NaCl (Beijing chemical works, Beijing, China) and 0.05% Tween 20 (Beijing Solarbio Science & Technology Co., Ltd., Beijing, China) pH 7.5) and incubated with appropriate dilutions of anti-OPN (1:4000) or anti-integrin β3 (1:3000) antibodies overnight at 4 °C. The next day, the membranes were washed three times with PBS and incubated with horseradish peroxidase-conjugated antibody against rabbit or mouse IgG (Boster, Wuhan, China) for 2 h at room temperature. After washing, the immunoreactive protein bands were visualized using an electrochemiluminescence detection kit (Thermol Biotech Inc., Rockford, IL, USA). Each experiment was repeated three times. The OD was analyzed on a gel image analysis system. The levels of OPN and integrin β3 were determined by calculating the OD ratio of OPN/β-actin and integrin β3/β-actin.

### 3.5. Genomic DNA Isolation, Bisulfite Modification of DNA and Methylation Specific PCR (MSP)

Genomic DNA was extracted from tissues with SDS/proteinase K treatment followed by phenol-chloroform extraction and ethanol precipitation. A total of 2 μg DNA from tissues was treated with NaOH (2 M) at 42 °C for 30 min then denatured at 95 °C for 5 min. Next, these DNA samples were incubated with 10 mM hydroquinone and 3.9 M sodium bisulfate (pH 5.0) at 54 °C for 16 h in the dark, purified using a Wizard DNA clean-up kit (Promega, Fitchburg, WI, USA), incubated with 3 M NaOH at 37 °C for 15 min and precipitated with 3 M ammonium acetate and 70% ethanol at −20 °C overnight. The next day, DNA samples were washed with 70% ethanol and dissolved in 15 μL TE buffer for PCR analysis of gene promoter methylation. PCR amplification was performed using 2.0 μL of bisulfite-modified DNA in a total volume of 25 μL reaction fluid containing 0.5 μL of each primer, 12.5 μL GC buffer, 4.0 μL dNTP mixture and 0.25 μL Takara LA Taq (Takara, Shiga, Japan). PCR conditions for OPN were 95 °C for 5 min, 40 cycles at 95 °C for 30 s, 56 °C for 30 s, and 72 °C for 45 s and then a final extension at 72 °C for 10 min. Conditions for integrin β3 were 95 °C for 5 min, 40 cycles of 95 °C for 30 s, 54 °C for 30 s, and 72 °C for 45 s and then a final extension at 72 °C for 10 min. The unmethylated primers for OPN were 5'-AGTAGTTGGGATTTAAGGTGTTT-3' (sense) and 5'-AAACACAATAACTCACACCTATA-3' (anti-sense), which produced a 163 bp band. The methylated OPN primers were 5'-AGTTGGGATTATAGGCGTTC-3' (sense) and 5'-CGCAATAACTCACGCCTATA-3' (anti-sense), which generated a 163 bp band. The unmethylated primers for integrin β3 were 5'-GGATTTGGAGTTGGTAAATGT-3' (sense) and 5'-AACTTCAACATCTCAAAAAACC-3' (anti-sense), which produced a 168 bp band. The methylated integrin β3 primers were 5'-ATTTGGAGTCGGTAAACGC-3' (sense) and 5'-AACTTCGACGTCTCGAAAAA-3' (anti-sense), which generated a 168 bp band. For MSP, DNA from placenta tissue was used as negative controls, and placenta tissue treated with Sss I (New England Biolabs Inc., Ipswich, MA, USA) was used as positive controls. The analysis was repeated three times.

### 3.6. Statistical Analysis

All statistical analyses were performed using SPSS 20.0 software (SPSS, Chicago, IL, USA). The Mann–Whitney *U*-test and Pearson chi-square test were used to generate *p* values; values less than 0.05 were considered statistically significant.

## 4. Conclusions

In this study, we identified methylation level of the promoter regions of OPN and integrin β3 and the expression of OPN and integrin β3 in varicosity. The methylation status negatively correlated with OPN and integrin β3 expression, which may be one of the reasons for the abnormal VSMCs phenotype switching and play an important role in the pathogenesis of varicosity. Because epigenetic mechanisms are reversible, DNA hypermethylation is expected to provide a new therapy for varicosity. Further *in vitro* and *in vivo* studies are warranted to elucidate the role and functions of DNA hypermethylation of OPN and integrin β3 in varicosity.

## References

[1] Nijsten T., van den Bos R.R., Goldman M.P., Kockaert M.A., Proebstle T.M., Rabe E., Sadick N.S., Weiss R.A., Neumann M.H. (2009). Minimally invasive techniques in the treatment of saphenous varicose veins. J. Am. Acad. Dermatol..

[2] Badier-Commander C., Couvelard A., Henin D., Verbeuren T., Michel J.B., Jacob M.P. (2001). Smooth muscle cell modulation and cytokine overproduction in varicose veins. An *in situ* study. J. Pathol..

[3] Gibbons G.H., Dzau V.J. (1994). The emerging concept of vascular remodeling. N. Engl. J. Med..

[4] Somers P., Knaapen M. (2006). The histopathology of varicose vein disease. Angiology.

[5] Majesky M.W. (2007). Developmental basis of vascular smooth muscle diversity. Arterioscler. Thromb. Vasc. Boil..

[6] Meda F., Folci M., Baccarelli A., Selmi C. (2011). The epigenetics of autoimmunity. Cell. Mol. Immunol..

[7] Kundu S., Peterson C.L. (1790). Role of chromatin states in transcriptional memory. Biochim. Biophys. Acta.

[8] Tazi J., Bird A. (1990). Alternative chromatin structure at CpG islands. Cell.

[9] Matouk C.C., Marsden P.A. (2008). Epigenetic regulation of vascular endothelial gene expression. Circ. Res..

[10] Turunen M.P., Aavik E., Yla-Herttuala S. (2009). Epigenetics and atherosclerosis. Biochim. Biophys. Acta.

[11] Friso S., Pizzolo F., Choi S.W., Guarini P., Castagna A., Ravagnani V., Carletto A., Pattini P., Corrocher R., Olivieri O. (2008). Epigenetic control of 11 β-hydroxysteroid dehydrogenase 2 gene promoter is related to human hypertension. Atherosclerosis.

[12] Ling C., Groop L. (2009). Epigenetics: A molecular link between environmental factors and type 2 diabetes. Diabetes.

[13] Alexander M.R., Owens G.K. (2012). Epigenetic control of smooth muscle cell differentiation and phenotypic switching in vascular development and disease. Annu. Rev. Physiol..

[14] Hu W.Y., Fukuda N., Satoh C., Jian T., Kubo A., Nakayama M., Kishioka H., Kanmatsuse K. (2000). Phenotypic modulation by fibronectin enhances the angiotensin II-generating system in cultured vascular smooth muscle cells. Arterioscler. Thromb. Vasc. Boil..

[15] Lesauskaite V., Epistolato M.C., Castagnini M., Urbonavicius S., Tanganelli P. (2006). Expression of matrix metalloproteinases, their tissue inhibitors, and osteopontin in the wall of thoracic and abdominal aortas with dilatative pathology. Hum. Pathol..

[16] Hoshikawa Y., Matsuda Y., Suzuki S., Okada Y., Tabata T., Matsumura Y., Kondo T. (2005). Osteopontin may be responsible for pulmonary vascular remodeling. Chest.

[17] Myers D.L., Harmon K.J., Lindner V., Liaw L. (2003). Alterations of arterial physiology in osteopontin-null mice. Arterioscler. Thromb. Vasc. Boil..

[18] Ye S., Sun Y., Bie A., Zhou Y., Liu J., Liu Q. (2009). Influence of osteopontin short hairpin RNA on the proliferation and activity of rat vascular smooth muscle cells. J. Huazhong Univ. Sci. Technol..

[19] Switala-Jelen K., Dabrowska K., Opolski A., Lipinska L., Nowaczyk M., Gorski A. (2004). The biological functions of β3 integrins. Folia Biol..

[20] Bendeck M.P., Irvin C., Reidy M., Smith L., Mulholland D., Horton M., Giachelli C.M. (2000). Smooth muscle cell matrix metalloproteinase production is stimulated via α_v_β_3_ integrin. Arterioscler. Thromb. Vasc. Boil..

[21] Bendeck M.P., Nakada M.T. (2001). The β3 integrin antagonist m7E3 reduces matrix metalloproteinase activity and smooth muscle cell migration. J. Vasc. Res..

[22] Han M., Wen J.K., Zheng B., Liu Z., Chen Y. (2007). Blockade of integrin β3–FAK signaling pathway activated by osteopontin inhibits neointimal formation after balloon injury. Cardiovasc. Pathol..

[23] Khan A.A., Eid R.A., Hamdi A. (2000). Structural changes in the tunica intima of varicose veins: A histopathological and ultrastructural study. Pathology.

[24] Thyberg J., Hedin U., Sjolund M., Palmberg L., Bottger B.A. (1990). Regulation of differentiated properties and proliferation of arterial smooth muscle cells. Arteriosclerosis.

[25] Wali M.A., Dewan M., Eid R.A. (2003). Histopathological changes in the wall of varicose veins. Int. Angiol..

[26] Elsharawy M.A., Naim M.M., Abdelmaguid E.M., Al-Mulhim A.A. (2007). Role of saphenous vein wall in the pathogenesis of primary varicose veins. Interact. Cardiovasc. Thorac. Surg..

[27] El-Hamamsy I., Yacoub M.H. (2009). Cellular and molecular mechanisms of thoracic aortic aneurysms. Nat. Rev. Cardiol..

[28] Sandbo N., Taurin S., Yau D.M., Kregel S., Mitchell R., Dulin N.O. (2007). Downregulation of smooth muscle α-actin expression by bacterial lipopolysaccharide. Cardiovasc. Res..

[29] Shanahan C.M., Weissberg P.L., Metcalfe J.C. (1993). Isolation of gene markers of differentiated and proliferating vascular smooth muscle cells. Circ. Res..

[30] Hultgardh-Nilsson A., Lovdahl C., Blomgren K., Kallin B., Thyberg J. (1997). Expression of phenotype- and proliferation-related genes in rat aortic smooth muscle cells in primary culture. Cardiovasc. Res..

[31] Findeisen H.M., Kahles F.K., Bruemmer D. (2013). Epigenetic regulation of vascular smooth muscle cell function in atherosclerosis. Curr. Atheroscler. Rep..

[32] Lim C.S., Davies A.H. (2009). Pathogenesis of primary varicose veins. Br. J. Surg..

[33] Mangiarua E.I., Galagedera N.J., Eastham L.L. (2001). Angiotensin II-induced growth effects in vascular smooth muscle in cell culture and in the aortic tunica media in organ culture. Arch. Physiol. Biochem..

[34] Zhang Y.M., Wang K.Q., Zhou G.M., Zuo J., Ge J.B. (2003). Endothelin-1 promoted proliferation of vascular smooth muscle cell through pathway of extracellular signal-regulated kinase and cyclin D1. Acta Pharmacol. Sin..

[35] Lagna G., Ku M.M., Nguyen P.H., Neuman N.A., Davis B.N., Hata A. (2007). Control of phenotypic plasticity of smooth muscle cells by bone morphogenetic protein signaling through the myocardin-related transcription factors. J. Biol. Chem..

[36] Weintraub A.S., Schnapp L.M., Lin X., Taubman M.B. (2000). Osteopontin deficiency in rat vascular smooth muscle cells is associated with an inability to adhere to collagen and increased apoptosis. Lab. Investig..

[37] Zohar R., Suzuki N., Suzuki K., Arora P., Glogauer M., McCulloch C.A., Sodek J. (2000). Intracellular osteopontin is an integral component of the CD44–ERM complex involved in cell migration. J. Cell. Physiol..

[38] Scatena M., Liaw L., Giachelli C.M. (2007). Osteopontin: A multifunctional molecule regulating chronic inflammation and vascular disease. Arterioscler. Thromb. Vasc. Boil..

[39] Giachelli C.M., Liaw L., Murry C.E., Schwartz S.M., Almeida M. (1995). Osteopontin expression in cardiovascular diseases. Ann. N. Y. Acad. Sci..

[40] Giachelli C.M., Bae N., Almeida M., Denhardt D.T., Alpers C.E., Schwartz S.M. (1993). Osteopontin is elevated during neointima formation in rat arteries and is a novel component of human atherosclerotic plaques. J. Clin. Investig..

[41] Coskun S., Atalar E., Ozturk E., Yavuz B., Ozer N., Goker H., Ovunc K., Aksoyek S., Kes S., Sivri B. (2006). Plasma osteopontin levels are elevated in non-ST-segment elevation acute coronary syndromes. J. Natl. Med. Assoc..

[42] Reik W. (2007). Stability and flexibility of epigenetic gene regulation in mammalian development. Nature.

[43] Robertson K.D. (2005). DNA methylation and human disease. Nat. Rev. Genet..

[44] Krishna S.M., Dear A., Craig J.M., Norman P.E., Golledge J. (2013). The potential role of homocysteine mediated DNA methylation and associated epigenetic changes in abdominal aortic aneurysm formation. Atherosclerosis.

[45] Zhang D., Wen X., Wu W., Xu E., Zhang Y., Cui W. (2013). Homocysteine-related hTERT DNA demethylation contributes to shortened leukocyte telomere length in atherosclerosis. Atherosclerosis.

[46] Webster A.L., Yan M.S., Marsden P.A. (2013). Epigenetics and cardiovascular disease. Can. J. Cardiol..

[47] Herman J.G., Baylin S.B. (2003). Gene silencing in cancer in association with promoter hypermethylation. N. Engl. J. Med..

[48] Clark S.J., Harrison J., Frommer M. (1995). CpNpG methylation in mammalian cells. Nat. Genet..

[49] Straussman R., Nejman D., Roberts D., Steinfeld I., Blum B., Benvenisty N., Simon I., Yakhini Z., Cedar H. (2009). Developmental programming of CpG island methylation profiles in the human genome. Nat. Struct. Mol. Boil..

[50] Park I.Y., Sohn B.H., Choo J.H., Joe C.O., Seong J.K., Lee Y.I., Chung J.H. (2005). Deregulation of DNA methyltransferases and loss of parental methylation at the insulin-like growth factor II (Igf2)/H19 loci in p53 knockout mice prior to tumor development. J. Cell. Biochem..

